# Endothelial cell transitions in zebrafish vascular development

**DOI:** 10.1111/dgd.12938

**Published:** 2024-07-27

**Authors:** Li‐Kun Phng, Benjamin M. Hogan

**Affiliations:** ^1^ Laboratory for Vascular Morphogenesis RIKEN Center for Biosystems Dynamics Research Kobe Japan; ^2^ Organogenesis and Cancer Programme Peter MacCallum Cancer Centre Melbourne Victoria Australia; ^3^ Sir Peter MacCallum Department of Oncology and the Department of Anatomy and Physiology University of Melbourne Melbourne Victoria Australia

**Keywords:** angiogenesis, endothelial cell transitions, lymphangiogenesis, zebrafish

## Abstract

In recent decades, developmental biologists have come to view vascular development as a series of progressive transitions. Mesoderm differentiates into endothelial cells; arteries, veins and lymphatic endothelial cells are specified from early endothelial cells; and vascular networks diversify and invade developing tissues and organs. Our understanding of this elaborate developmental process has benefitted from detailed studies using the zebrafish as a model system. Here, we review a number of key developmental transitions that occur in zebrafish during the formation of the blood and lymphatic vessel networks.

## Graphical abstract

The vertebrate vasculature is an elaborate, interconnected, hierarchical network of blood and lymphatic blood vessels found in all tissues and organs. The formation of functional vascular networks during embryonic development is a progressive process that first initiates with the formation of endothelial cells (ECs) that will line future vessels. In this review, we describe important transitions that occur during the formation of vascular lineages using the zebrafish as a model system.

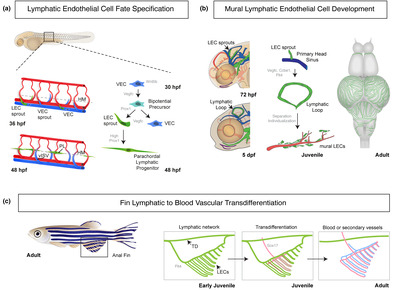

## 1 INTRODUCTION

The vertebrate vasculature is an elaborate, interconnected, hierarchical network of closed tubes found in all tissues and organs. Vasculature serves the physiological functions of carrying blood, tissue fluids, immune cells and nutrients around the body. The formation of functional vascular networks during embryonic development is a progressive process that first initiates with the formation of endothelial cells (ECs) that will line future vessels. Vascular development progresses with the expansion of the vascular tree and involves the maturation of vessels, including the formation of mural cell populations and vascular smooth muscle (Potente & Makinen, 2017).

There are two main vascular networks: the blood vessels, that form first via vasculogenesis and angiogenesis; and the lymphatic vessels, that form later by lymphangiogenesis. In recent decades, cell and developmental biologists have come to view vascular development as a series of stepwise transitions that include changing early mesodermal progenitors to ECs, diversifying EC fates and transitioning through a series of cell behaviors that are essential to ultimately build mature blood and lymphatic vessel networks. These transitions have been well described in many vertebrate model organisms, but the zebrafish model has provided a deep new understanding of many key transitions that drive vascular development (for review, see Hogan & Schulte‐Merker, 2017). In this review, we focus on a series of transitions that occur during the formation of vascular lineages and that have been described using the zebrafish as a model system. We cover both the formation of blood vascular and lymphatic vascular lineages, as well as highlighting unexpected transitions by which vasculature can produce diverse new cell types.

## 2 FORMATION OF THE FIRST EMBRYONIC VESSELS: DIFFERENTIATION OF ANGIOBLASTS TO ENDOTHELIAL CELLS

The first vessels that form during embryogenesis are the axial vessels – the dorsal aorta (DA) and cardinal vein (CV) – that span along the anterior‐to‐posterior axis of the embryonic trunk to establish the first circulatory loop in the embryo. The axial vessels are generated de novo from angioblasts, a process known as vasculogenesis. At around 12 h post fertilization (hpf), cells at the lateral plate mesoderm (LPM) become specified as angioblasts. The basic‐helix–loop–helix‐PAS transcription factor, Npas4l, is the master regulator of the endothelial lineage that induces the expression of *etv2/etsrp*, an early endothelial lineage marker, as well as *scl/tal1*, an early hematopoietic lineage marker (Reischauer et al., 2016). After specification, angioblasts migrate from the LPM towards the embryonic midline, where they coalesce and assemble to form the DA and posterior CV (PCV) beneath the notochord by 17–19 hpf (Jin et al., 2005). The process of guided angioblast migration depends on etv2/etsrp function (Sumanas & Lin, 2006), Elabela/Apelin‐Apelin receptor signaling (Helker et al., 2015) and on the creation of a cavity at the midline through somite morphogenesis (Paulissen et al., 2022). The exact mechanism of how DA and PCV are established has been debated. In one model, angioblasts originate from a common location in the LPM, coalesce to form a single vascular cord at the position of the DA and undergo ventral sprouting, giving rise to the PCV. In this model, vascular endothelial growth factor a (Vegfa)‐induced *efnb2a* expression in the vascular cord and bidirectional Ephb4a‐Efnb2a signaling promote the retention of arterial angioblasts in the DA and ventral sprouting of venous angioblasts to form the CV (Herbert et al., 2009). Another model proposes that two distinct angioblasts – medial and lateral – exist at the LPM. The medial angioblasts migrate to the midline first and contribute almost exclusively to the DA, whereas lateral angioblasts start to migrate later and give rise to the PCV (Kohli et al., 2013). It may be that both cellular mechanisms are involved in the initial separation of the DA and PCV to different degrees. However, the use of direct lineage tracing by Kohli et al. (2013) suggests that a significant degree of angioblast separation is already present during the migration of angioblasts from the LPM to the midline. Studies that image this transition with increasing resolution are needed to fully understand the relative importance and timing of key angioblast contributions.

The differentiation of angioblasts into ECs of arterial and venous identity is genetically determined. Vegfa activity and SoxF transcription factors act upstream of the Notch pathway to specify arterial identity. Disruption of Vegfa and Notch signaling leads to a loss of artery‐specific markers and ectopic expression of venous markers within the DA (Lawson et al., 2001; Lawson et al., 2002). SoxF proteins directly modulate the activity of arterial‐specific enhancers for Notch1b (Chiang et al., 2017) and the combined loss of *sox7* and *sox18* expression results in severe loss of arterial identity of the presumptive aorta (Cermenati et al., 2008; Herpers et al., 2008; Pendeville et al., 2008). Venous identity also depends on Notch signaling, which suppresses venous cell markers in the DA (Lawson et al., 2001), and the orphan nuclear receptor Coup‐TFII (*nr2f2*), whose activity induces the expression of venous genes and the proper differentiation of the CV (Swift et al., 2014).

In addition to LPM‐derived angioblasts, additional sources of endothelial progenitor cells contribute to axial artery and vein formation. Recent studies have identified a population of precursors called somite‐derived endothelial cells (SDECs) as another source of DA endothelium. SDECs start to appear at the 12‐somite stage, delaminate from the dermomyotome, and migrate to and incorporate into the developing DA (Nguyen et al., 2014; Sahai‐Hernandez et al., 2023). A cellular source of progenitors EC has also been detected in the zebrafish tail. From 16 hpf, *sox32* ^+^ *sox17* ^+^ endodermal cells located bilateral to the Kupffer's vesicle differentiate into *islet1* (*isl1*)^+^ endothelial progenitors in a Bmp/Smad signaling‐dependent manner (Nakajima et al., 2023). The *ist1* ^+^ endothelial progenitors subsequently differentiate through the Npas4l‐Etv2/Etsrp signaling pathway into caudal venous ECs, with 80% of these cells subsequently forming the caudal hematopoietic tissue (Nakajima et al., 2023). Overall, multiple cell types can contribute to different vascular lineages and suggest an intrinsic plasticity that remains to be fully understood in the transitions that drive vascular development.

## 3 SPROUTING ANGIOGENESIS IN EARLY EMBRYOGENESIS: SPECIFICATION OF ENDOTHELIAL TIP AND STALK CELLS

**FIGURE 1:**
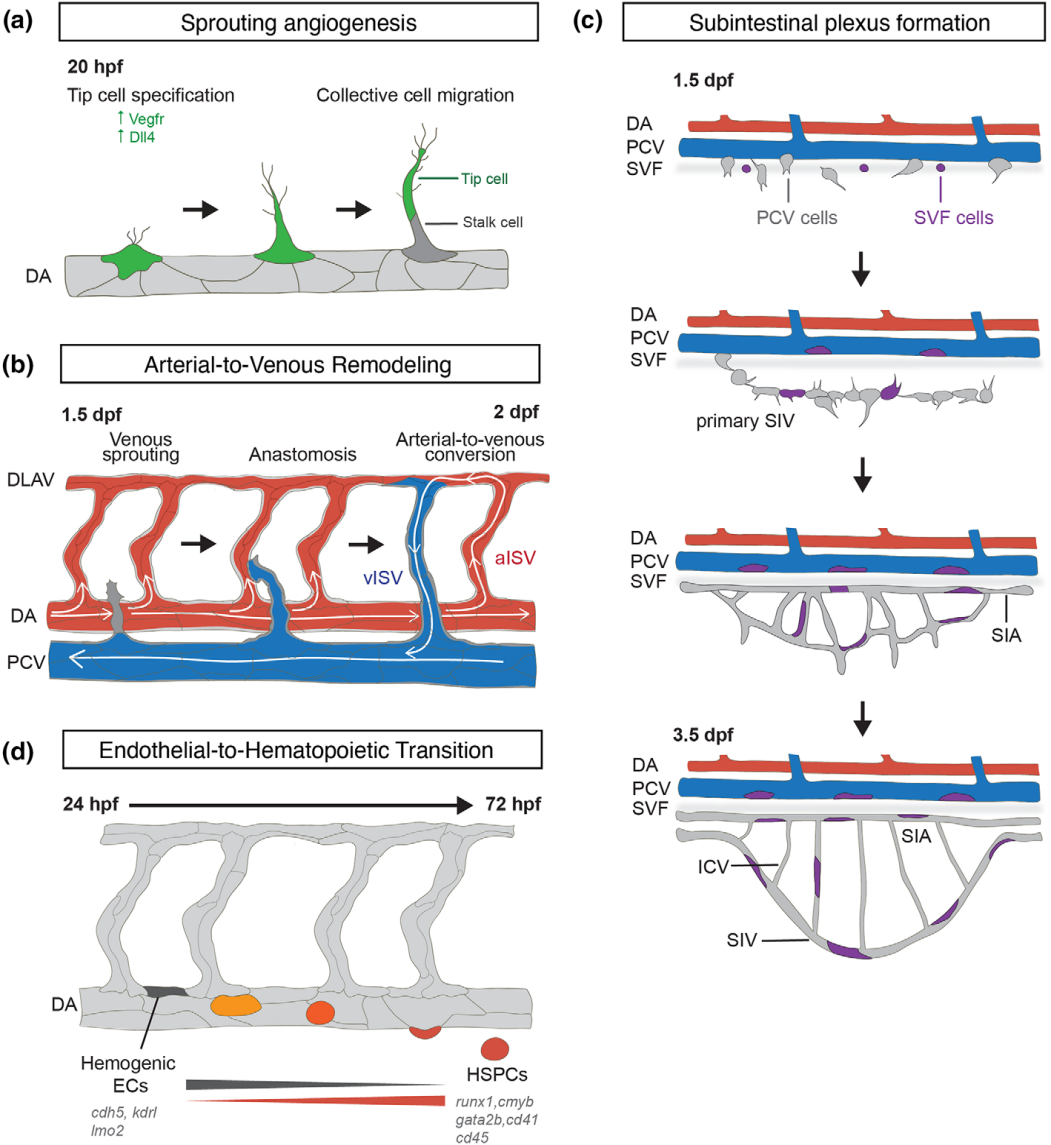
Building blood vascular networks through transitions in endothelial cell (EC) fates and behaviors. (a) Formation of intersegmental vessels (ISVs) through sprouting angiogenesis. Quiescent ECs in the dorsal aorta (DA) become specified as tip cells to spearhead the formation of new vascular sprouts. hpf, hours post fertilization. (b) Arterial‐to‐venous remodeling of ISVs. Around 50% of arterial ISVs (aISVs) connect to the posterior cardinal vein (PCV), so becoming venous ISVs (vISVs). DLAV, dorsal longitudinal anastomotic vessels. (c) ECs derived from the PCV (gray) and vascular progenitor cells (purple) from the second vascular field (SVF) contribute to the formation of subintestinal plexus. SIA, subintestinal artery; SIV, subintestinal vein; ICV, interconnecting vessel; dpf, days post fertilization. (d) Endothelial‐to‐hematopoietic (EHT). Hematopoietic stem and progenitor cells (HSPCs, red) are generated by specialized hemogenic ECs (dark gray) in the DA.

New blood vessels are also formed through the expansion of existing blood vessels by sprouting angiogenesis. Soon after the formation of the DA, at around 22 hpf, a subset of ECs become specified as tip cells at each somite boundary to initiate the formation of primary intersegmental vessels (ISVs, Figure 1a) (Isogai et al., 2003). The tip cell is highly invasive, generating many membrane protrusions such as filopodia and lamellipodia. Tip cells undergo extensive cell shape changes that drive their emergence from the DA and migration towards the dorsal roof of the neural tube, where they branch out to form the dorsal longitudinal anastomotic vessel (DLAV). Trailing tip cells are the stalk cells and, together, they migrate collectively to drive vascular sprout elongation. A later wave of secondary sprouting occurs from the PCV at 30–32 hpf to form venous ISVs or lymphatic vessels and is described in further detail below.

The specification of tip cells during sprouting angiogenesis is tightly controlled and has been extensively reviewed in recent years (Cuervo et al., 2023; Hogan & Schulte‐Merker, 2017; Siekmann et al., 2013). Briefly, the formation of tip cells from the DA is controlled by Vegfa‐Kdr/Kdrl and Notch signaling pathways (Covassin et al., 2006; Covassin et al., 2009;Leslie et al., 2007; Siekmann & Lawson, 2007). Vegfa induces *dll4* expression in an extracellular signal‐regulated kinase‐dependent manner (Shin, Beane, et al., 2016) and ECs with the highest Vegfa stimulation and Dll4 expression become specified as tip cells. Dll4‐expressing tip cells activate Notch signaling in stalk cells to prevent the formation of more tip cells (a process known as lateral inhibition). The inhibition of Notch signaling leads to ectopic tip cell specification and increased EC proliferation, thereby causing hypersprouting and aberrant vascular network formation with a disturbed flow pattern (Leslie et al., 2007; Siekmann & Lawson, 2007). However, not all sprouting events are stimulated by Vegfa signaling. In the zebrafish trunk, sprouting of venous ECs from the PCV to generate venous or lymphatic ISVs is dependent on Vegfc signaling (Hogan et al., 2009), while the formation of the caudal vein plexus through new sprouts from the caudal vein is dependent on Bmp signaling (Wiley et al., 2011). In the brain, Gpr124‐ and Reck‐dependent Wnt/beta‐catenin signaling promotes tip cell formation and function to form central arteries by expressing MMP25, which confers brain invasive competence by cleaving collagen IV in the pial basement membrane (Schevenels et al., 2024; Vanhollebeke et al., 2015). Hence, depending on the vascular bed, different signals activate quiescent ECs of a pre‐existing vessel to induce the formation of more invasive endothelial tip cells that spearhead the formation of new blood vessels.

## 4 REMODELING OF BLOOD VESSELS: FROM ARTERIES TO VEINS

Following the establishment of a trunk vascular network consisting of ISVs that connect the DA with the DLAV, remodeling of ISVs occurs where approximately half of ISVs lose their arterial identity to generate a balanced number of arterial ISVs (aISVs) and venous ISVs (vISVs) (Bussmann et al., 2010; Isogai et al., 2003). vISVs are generated through the disconnection of the primary ISV from the DA and its connection with a vascular sprout arising from the PCV, providing a venous connection and drainage of blood flow (Figure 1b). Therefore, although all primary ISVs are of arterial origin, there is a subsequent conversion to venous function and the formation of a balanced number of arteries and veins.

The mechanism by which ISVs remain as arteries or remodel to veins remains controversial. Early data show that the pattern of aISVs and vISVs is regulated by Notch signaling (Geudens et al., 2010). Dll4 loss of function led to all ISVs forming vISVs at the expense of aISVs. It seems likely that ISV fate is dictated by the decision of venous cells to anastomose to aISVs and that this is controlled by Notch signaling (Geudens et al., 2010). Two recent studies further implicate the roles of blood flow and Notch signaling in regulating arterial–venous patterning of ISVs (Geudens et al., 2019; Weijts et al., 2018). Although both studies demonstrate that Notch signaling maintains the arterial identity of primary ISVs, the mechanism by which it does so is conflicting. Weijts et al. (2018) propose that the onset of blood flow in ISVs activates Notch signaling in ECs, which then prevents the anastomosis of secondary sprouts from the PCV to the ventral segment of the ISV. Anastomosis of secondary sprouts, however, can occur in neighboring ISVs with lower blood flow, thereby transforming them into veins. Venous blood flow subsequently induces dorsal (or upstream) migration of venous ECs from the PCV, leading to the displacement of the original arterial ECs. On the contrary, work by Geudens et al. (2019) suggests that primary ISVs are pre‐specified in a Notch‐dependent process independent of connections with venous sprouts from the PCV and blood flow. They observed that ECs of future aISVs and vISVs already show differential cell polarity, directional migration and ventral junction organization (unicellularity versus multicellularity) before sprouting and anastomosis. In one elegant experiment, it was shown that when all the venous sprouting from the PCV is blocked (in the absence of Ccbe1), aISVs still disconnect from the DA. Hence, ECs of future vISVs are hardwired to migrate dorsally even when a secondary sprout from the PCV does not connect to the ISV (Geudens et al., 2019). Given these findings, the authors propose that blood flow functions to fine‐tune the global balance of arteries and veins, but that the initial decision is hardwired by cell intrinsic factors in the trunk vasculature.

## 5 GENERATION OF ORGAN‐SPECIFIC VASCULATURES

ECs generally adopt a more quiescent behavior after new blood vessels develop lumens and become perfused. However, they can be reactivated to form new vascular sprouts and generate new vascular networks in neighboring tissues or to generate organ‐specific vessels. This is observed during the formation of the subintestinal plexus, which later gives rise to the liver and pancreatic vasculature (Hen et al., 2015). The subintestinal plexus develops on both sides of the yolk ball and resembles a vascular basket composed of subintestinal vein (SIV), subintestinal artery and interconnecting vessels (Figure 1c) (Isogai et al., 2001). Time‐lapse live imaging and lineage tracing experiments demonstrate that ECs at the ventral portion of the PCV contribute to the formation of vessels of the subintestinal plexus (Goi & Childs, 2016; Hen et al., 2015; Koenig et al., 2016). From 28 to 32 hpf, these so‐called ventral PCV angioblasts sprout from the PCV, migrate ventrally over the yolk ball and anastomose with adjacent sprouts to form the primary SIV. ECs in the SIV subsequently migrate dorsally to form the subintestinal artery in a Vegfa‐dependent manner, and ventrally to form SIV sprouts in a Bmp‐dependent manner, creating vascular loops in the process. The subintestinal plexus expands through ventral migration of ECs in sprouts (or leading buds) and remodels through vascular pruning and retraction of SIV sprouts (Goi & Childs, 2016; Hen et al., 2015; Lenard et al., 2015). A more recent work further revealed a different cellular origin of the subintestinal plexus. After 24 hpf, *etv2/etsrp* ^+^ and *tal1* ^+^ vascular progenitor cells emerge from the secondary vascular field (SVF), a region along the yolk extension, and incorporate into the existing PCV as well as contributing directly to the formation of the subintestinal vasculature (Figure 1c), (Metikala et al., 2022). The inhibition of SVF differentiation resulted in defective formation of subintestinal vasculature, particularly the subintestinal artery. Notably, SVF cells participate in vascular recovery after chemical ablation of vascular ECs in trunk vessels (Metikala et al., 2022). This uncovers an example of a unique vascular field needed to generate an organ‐specific vessel network. It will be interesting to determine if similar, separate vascular fields produce organ‐specific vasculatures at later stages of development.

## 6 VESSEL ARTERIALIZATION DURING ORGANOGENESIS: VENOUS‐TO‐ARTERIAL TRANSITION

During early embryogenesis such as the development of ISVs, arterial ECs form aISVs and venous ECs generate vISVs. However, it is now well‐appreciated that sprouting frequently occurs from veins to generate arteries at later stages of organogenesis (Red‐Horse & Siekmann, 2019), highlighting the plasticity of EC fate. The formation of vein‐derived arteries has been observed in the central nervous system as well as during fin regeneration. The hindbrain vasculature consists of a centrally located basilar artery that connects to the bilateral primordial hindbrain channels (PHBCs) by arch‐shaped central arteries (Bussmann et al., 2011; Fujita et al., 2011). Both the basilar artery and central arteries are derived from PHBCs, which are of venous origin, through Notch‐dependent sprouting angiogenesis. Unlike secondary sprouting in the trunk vessels to generate vISVs, sprouting from the PHBCs is regulated by Vegfa‐Kdrl and Cxcl12b‐Cxcr4a signaling (Bussmann et al., 2011). The formation of ocular vasculature also requires sprouting of ECs from the venous primordial midbrain channel to form the nasal ciliary artery that connects with the cranial division of the carotid artery, thereby creating a functional artery–venous flow circuit (Isogai et al., 2001; Kochhan et al., 2013). The Notch pathway is again required in the generation of this plexus, with tip cells first expressing *dll4* followed by Notch pathway activation (Hasan et al., 2017). Additionally, Notch activation triggers the expression of the chemokine receptor *cxcr4a*, whose function is required for the migratory activity of nasal ciliary artery tip cells (Hasan et al., 2017). The contribution of venous ECs to developing arteries is also observed in fin regeneration in adult zebrafish. Live imaging revealed that blood vessels regenerate through active proliferation of venous ECs, which migrate more extensively towards the expanding vascular front. Notably, these vein‐derived endothelial tip cells subsequently change their direction of movement to migrate against the expanding vascular front and blood flow to contribute to developing arteries (Xu et al., 2014). Again, Cxcr4a‐Cxcl12a signaling is necessary for proper arterial morphogenesis during fin regeneration, and it may be that this signaling axis is a common player when venous‐derived arterialization occurs during development.

## 7 DEFINITIVE HEMATOPOIESIS: ENDOTHELIAL‐TO‐HEMATOPOIETIC TRANSITION

The cellular constituents of blood – erythrocytes and the lympho‐myeloid cells of the immune system – are produced and replenished by hematopoietic stem and progenitor cells (HSPCs) throughout life (Jagannathan‐Bogdan & Zon, 2013). In the zebrafish, HSPCs are derived from the DA during the definitive wave of hematopoiesis, between 24 and 72 hpf, in a process known as endothelial‐to‐hematopoietic transition (EHT, Figure 1d) (Bertrand et al., 2010; Kissa et al., 2008; Kissa & Herbomel, 2010; Lam et al., 2010; Murayama et al., 2006). For more details about genetic and extracellular cues regulating HSPC emergence, we would like to refer readers to recent reviews on this topic (Sugden & North, 2021; Yvernogeau et al., 2023). Briefly, EHT is defined by transcriptional and morphological transitions in hemogenic ECs located in the DA. Initially, ECs express high levels of endothelial genes such as *kdrl*, *cdh5*, and *lmo2*. As EHT progresses, hemogenic ECs gradually lose their endothelial identity and instead elevate the expression of genes such as *runx1*, *cmyb*, *gata2*, and *cd45*, markers of definitive hematopoiesis commitment (Bertrand et al., 2010; Kissa et al., 2008; Lam et al., 2010). Time‐lapse live imaging has been instrumental in revealing the morphodynamics of ECs undergoing EHT. First, unlike in other vertebrate species where HSPCs form intra‐aortic hematopoietic clusters of cells that are released into the DA lumen, HSPCs in zebrafish extrude from the ventral wall of the DA as single cells into the subaortic space. Second, hemogenic ECs arise at the dorsal roof of the DA but migrate to the ventral floor plate, where extrusion takes place during EHT (Campinho et al., 2020; Kissa & Herbomel, 2010). Third, hemogenic ECs undergo significant morphological transitions in cell shape, from a flattened morphology to one that is rounded, and at the point of extrusion, a cup‐shaped cell. High‐resolution imaging showed inward bending of the apical (luminal) membrane during extrusion, anisotropic organization of actin cytoskeleton and tight junctions, and pulsatile characteristic of apical constriction of the cell that culminates in the release of the HSPC from the DA wall (Lancino et al., 2018). The HSPCs subsequently transmigrate through the PCV to enter the circulation and seed the caudal hematopoietic tissue in the tail region of the embryo, eventually populating the thymus and kidney (Kissa et al., 2008; Kissa & Herbomel, 2010; Lam et al., 2010). Lineage tracing experiments show that ECs originating from the DA give rise to leukocytes and myeloid cells in whole kidney marrow, demonstrating a robust, multilineage, long‐term population of the adult hematopoietic organ (Bertrand et al., 2010). This is an example of ECs transdifferentiating into a completely new lineage and is a critical transition during the formation of the blood system.

## 8 TRANSITIONS DURING LYMPHATIC VASCULAR DEVELOPMENT AND SPECIALIZATION

The lymphatic vasculature is a vertebrate‐specific vascular network. Best characterized in mammals, it is made up of blind‐ended capillary beds in peripheral tissues that connect to collecting lymphatic vessels and ultimately drain into the blood vasculature at the lympho‐venous valves. This complex anatomical network facilitates tissue fluid drainage, the trafficking of innate and adaptive immune cells, as well as having specialized organ‐specific functions (for review, see Oliver et al., 2020). Given the fundamental physiological importance of this system, lymphatics contribute to a number of diseases that include congenital disorders (such as lymphedema and lymphatic malformation), cancer and cardiovascular disease (Oliver et al., 2020). The formation of this second vasculature occurs via a series of well‐studied developmental transitions in vertebrate embryos (Koltowska et al., 2013). Here, we summarize key transitions that have been well studied in development of the zebrafish.

## 9 LYMPHATIC ENDOTHELIAL CELL FATE SPECIFICATION: THE TRANSITION FROM BLOOD TO LYMPHATIC VASCULATURE

**FIGURE 2:**
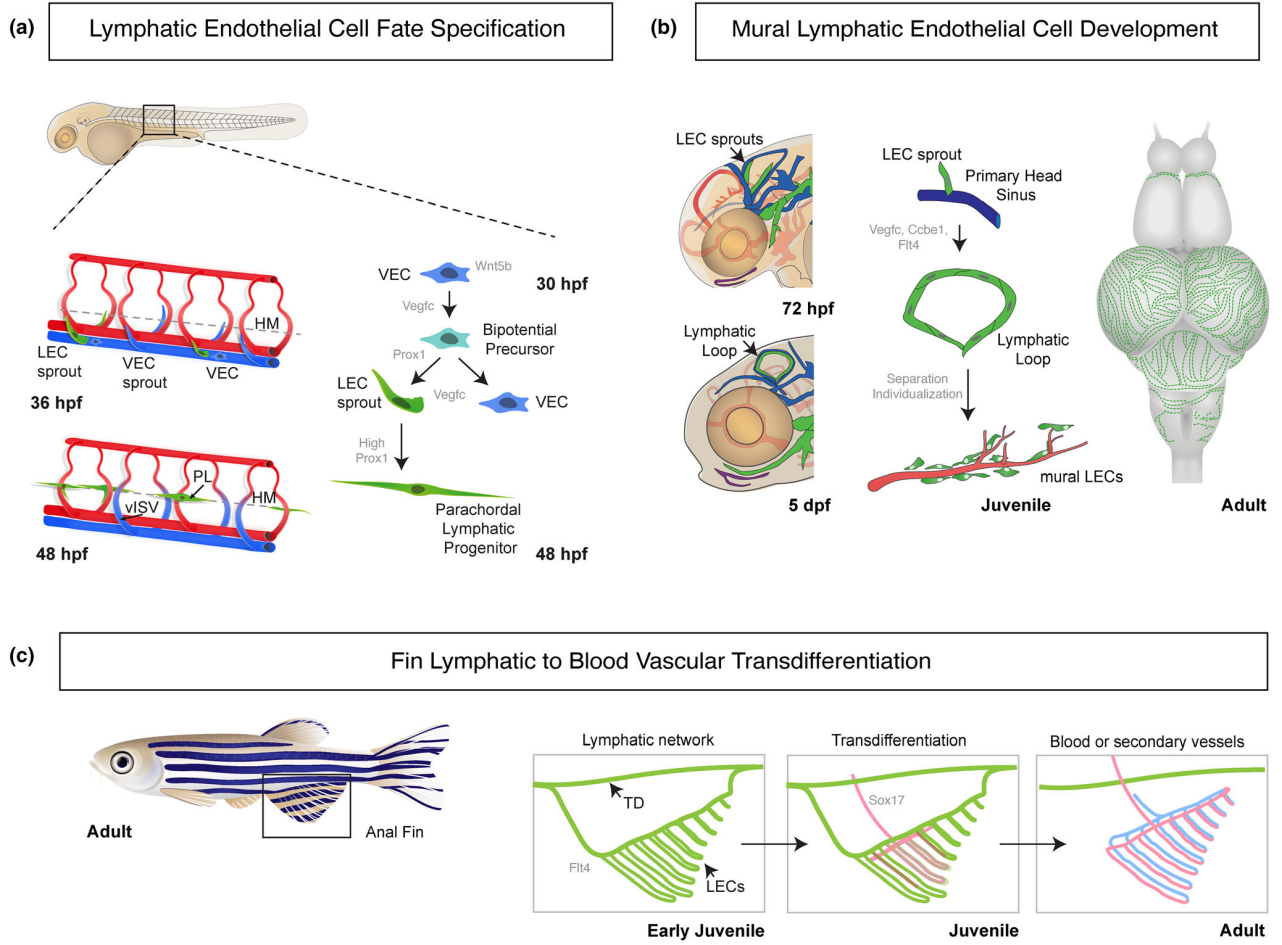
Transitions in development of lymphatic vasculature. (a) Schematic summary of lymphatic endothelial cell fate specification. (Left) LEC sprouts express Prox1 (green) and depart the PCV concomitant with VEC sprouts (blue) to migrate dorsally to the HM, while VEC sprouts anastomose with adjacent aISVs for form vISVs. (Right) Wnt5b is known to regulate cell divisions in the early PCV, Vegfc controls Prox1 expression and cell division of early bipotential progenitors in the PCV. Prox1 expression is initially low but increases to higher levels as cells sprout from the PCV to the HM. (b) Schematic summary of mural lymphatic endothelial cell development. Sprouting of LECs from the PHS at 72 hpf is regulated by Flt4, Ccbe1, and Vegfc and gives rise to the lymphatic loop (LL) by 5 dpf. Subsequently, mural LECs disperse into individual cells that are mural to blood vessels and ultimately cover the surface of the adult brain (right). (c) Schematic summary of lymphatic to blood vascular transdifferentiation in the anal fin (AF). Location of the AF in an adult animal (left). Initially a lymphatic network (green) forms in the AF that is connected to the thoracic duct (TD) and its development is dependent on Flt4. At juvenile stages, Sox17‐expressing cells (red) appear and LECs co‐express Sox17 as they begin to transdifferentiate. By adult stages the AF has a low flow blood vasculature including arteries (red) and veins (blue) with lymphatics absent having been replaced by blood vessels. aISV, arterial intersegmental vessel; Dpf, days post fertilization; Hpf, hours post fertilization; LEC, lymphatic endothelial cell; LL, lymphatic loop; PCV, posterior cardinal vein; PHS, primary head sinus; VEC, venous endothelial cell; vISV, venous intersegmental vessel.

The lymphatic vasculature forms through a highly conserved process whereby lymphatic vessels derive primarily from the CV in the early embryo. Lymphatics form from a limited number of specified lymphatic endothelial cell (LEC) progenitors initially found in the walls of functional CV (Hogan et al., 2009; Koltowska, Lagendijk, et al., 2015; Kuchler et al., 2006; Nicenboim et al., 2015; Shin, Male, et al., 2016; Yaniv et al., 2006). The zebrafish PCV undergoes a morphological change preceding the first specification of LEC progenitors. The vein becomes structurally polarized between 24 and 30 hpf, with increasing numbers of ECs accumulating at the dorsal side of the vessel over this time (Koltowska, Lagendijk, et al., 2015). This involves a ventral to dorsal movement of venous ECs, coupled with the proliferation of these cells (Koltowska, Lagendijk, et al., 2015; Nicenboim et al., 2015). The specification of the lymphatic lineage is governed by the transcription factor Prox1, which is both necessary and to some degree sufficient to transdifferentiate venous ECs into LECs in vertebrates (Hong et al., 2002; Kim et al., 2010; Srinivasan & Oliver, 2011; Wigle & Oliver, 1999). Endogenous Prox1a protein expression has been analyzed to provide a clear picture of the timing and localization of LEC specification in zebrafish (Baek et al., 2019;Koltowska, Lagendijk, et al., 2015; Shin, Male, et al., 2016). Prox1 expression initiates in the PCV between 30 and 32 hpf. Lymphangiogenesis proceeds with the dorsal sprouting of these Prox1‐positive progenitors from the PCV, which occurs during secondary angiogenesis (Figure 2a). The Prox1‐positive LEC progenitors in the PCV undergo a cell division, with one daughter cell remaining in the PCV and the other migrating dorsally from the PCV to generate an LEC. Hence, LEC progenitors in the zebrafish trunk are bipotential (Koltowska, Lagendijk, et al., 2015) (Figure 2a). It remains unclear if molecular asymmetry is present at the bipotential precursor stage or following their division, although Prospero (the Drosophila homologue of Prox1) has been shown to act in true asymmetric divisions in neuroglioblast development in the fly (Hirata et al., 1995). Interestingly, in mammals there have been recent reports of non‐venous origins of LECs during development (Klotz et al., 2015; Mahadevan et al., 2014; Martinez‐Corral et al., 2015; Stanczuk et al., 2015; Stone & Stainier, 2019) and in zebrafish using direct live imaging, it was elegantly shown that the development of the facial LECs involved contributions from ECs of non‐venous origins (Eng et al., 2019). This work may indicate the involvement of heterogeneous origins for LECs during development, although it is clear that the majority of LECs originate from veins in zebrafish development.

Recent work has investigated the molecular mechanism by which Prox1 directs lymphatic fate in the zebrafish. A single cell sequencing‐based analysis of multiple stages of lymphangiogenesis revealed the transcriptional changes that accompany the transition from venous EC (VEC) to LEC (Grimm et al., 2023). In a genetic model in which Prox1 function was depleted (in *prox1a* zygotic mutants) mutant ECs undergo a progressive reversion from LECs to VECs. In this setting, an up‐regulation of blood vascular and hematopoietic transcriptional programmes was concomitant with a loss of LEC marker genes. Furthermore, in mutants null for embryonic Prox1 homologue function (*prox1a/1b* maternal and zygotic mutants), there was an up‐regulation of blood vascular and hematopoietic marker genes at stages when the first LECs would be departing the PCV. This work suggested that Prox1 initiates LEC fate largely by down‐regulating blood vascular and hematopoietic pathways (Grimm et al., 2023). Further studies are needed to understand the key functional targets of Prox1 and how they cooperate to direct lineage specification.

The transition to an LEC from a blood vascular EC takes place concomitant with the sprouting that occurs during secondary angiogenesis (described above). Prox1‐positive LECs sprout from the PCV at the same time as venous sprouts emerge (Koltowska, Lagendijk, et al., 2015; Shin, Male, et al., 2016). LECs migrate to the horizontal myoseptum (HM) to form parachordal lymphatic progenitors (PLs); however, on average, only every second sprout from the PCV will form a PL and every other sprout will anastomose to an adjacent aISV and form a vISV (Bussmann et al., 2010) (Figure 2a). It has been hypothesized that a key decision‐making step in LEC specification in the zebrafish trunk was the decision of the venous sprout to connect with an adjacent aISV, or not to connect (as described above). However, an emerging model suggests that Notch signaling is involved in hardwiring ISV fate (Geudens et al., 2019) and importantly, the initiation of Prox1 expression in the PCV precedes cell sprouting. Together, this suggests that the fate of LECs is largely hardwired and the right balance of aISVs, vISVs, and PLs is therefore established early, by key molecular determinants in each lineage.

LEC sprouting from the PCV to the HM is dependent on Vegfc‐Flt4 signaling. Vegfc is necessary to induce detectable levels of Prox1 in the PCV and to drive the progressive up‐regulation of Prox1 (consolidating LEC fate) (Koltowska, Lagendijk, et al., 2015; Shin, Male, et al., 2016). The further migration of specified LECs through the embryo occurs along arterial ECs, mesenchymal fibroblasts, and pericytes and is regulated by a pathway of Vegfc, Ccbe1, and Adamsts3 that is laid down by multiple cellular sources (Peng et al., 2022; Wang et al., 2020). Chemokine signaling (through cxcr4, cxcl12 homologues) controls the ongoing migration of LECs as they depart the HM, migrate along arterial substrates and contribute to the thoracic duct and dorsal longitudinal lymphatics (Cha et al., 2012). Netrins and collagens also play a role in controlling the migratory environment (Chaudhury et al., 2020; Navankasattusas et al., 2008). Within the LEC itself, there is evidence that Notch1b is essential but acts after the normal induction of Prox1 expression (Grimm et al., 2023). A number of transcription factors have also been implicated in the regulation of lymphangiogenesis with Mafba, Sox18, Nfatc1, and CoupTF2 loss of function models displaying phenotypes in lymphatic development (Aranguren et al., 2011; Jung et al., 2019; Koltowska, Paterson, et al., 2015; Kulkarni et al., 2009; van Impel et al., 2014). Based on knowledge from mouse models it seems likely that these factors may influence the fate of LECs, but this remains to be definitively demonstrated in zebrafish. At the end of this process, between 3 and 4 dpf, the first lumenized lymphatic vessels form in the embryo, ending the transition from blood to lymphatic vasculature.

## 10 THE TRANSITION OF LECs TO LYMPHATIC VALVES IN ZEBRAFISH

One major feature of lymphatic vessels in mammals is the presence of lymphatic valves. Lymphatic valves ensure the unidirectional drainage of lymph within lymphatics. Previous studies have noted the absence of lymphatic valves in fish (Kampmeier, 1969). In the absence of valves, higher peripheral tissue pressure in fish and contraction of skeletal muscle could ensure unidirectional movement of lymph within vessels. However, a recent study has now demonstrated that zebrafish are not entirely devoid of lymphatic valves. Shin et al. (2019) explored the facial lymphatic structures of the zebrafish larvae using confocal imaging, functional lymphangiography, and extensive electron microscopy to identify a single lymphatic valve separating the lateral facial lymphatic from a large rostral lymphatic structure that sits lateral to the primary head sinus (PHS) (Shin et al., 2019). In this study, it was shown using live imaging of a *gata2* enhancer reporter transgenic line that future valve ECs originate in the primary head sinus and condense and arrange into lymphatic valves over ~2 days of development. Indicative of this valve being a conserved feature of vertebrate lymphatics, Gata2 is an established marker of developing lymphatic valves in mice and essential for valve formation in humans (Kazenwadel et al., 2012; Kazenwadel et al., 2015; Mahamud et al., 2019; Shin et al., 2019). Furthermore, this study uncovered that *gata2*, *prox1*, and *itga5* are all functionally essential for the formation of mature lymphatic valves with wild‐type valve leaflets. These genes are also all essential in mice (for review, see Koltowska et al., 2013). Taken together, the transition from LECs in lymphatic vessel walls to lymphatic valves appears to be a conserved feature in zebrafish.

## 11 AN UNEXPECTED TRANSITION AT BRAIN BORDERS: THE FORMATION OF MURAL LYMPHATIC ENDOTHELIAL CELLS

The vertebrate brain is devoid of lymphatic vessels. This is likely to maintain cerebrospinal fluid homeostasis and immune privilege. In 2017, a surprising discovery of a new lymphatic cell type was made by three different laboratories (Bower et al., 2017; van Lessen et al., 2017; Venero Galanternik et al., 2017). A population of LECs of unique and unusual morphology were identified adjacent to the meninges within the brain of larval, juvenile, and adult zebrafish. These LECs emerge from the PHS in development and sprout dorso‐laterally to form an initial loop of Prox1‐, Mrc1‐ and Lyve1‐positive LECs in the midbrain of the developing embryo by ~3 dpf (Figure 2b). The development of this structure is dependent on Prox1, Vegfc, Flt4 and Ccbe1 activity during development and transcriptomics revealed that these cells are LECs or very closely related to LECs (Bower et al., 2017; van Lessen et al., 2017; Venero Galanternik et al., 2017). Remarkably, following the formation of this LEC loop at early stages, these cells progressively dissociate from each other, become more mesenchymal in appearance and eventually form a population of isolated individual cells present throughout the meninges (Figure 2b). They are found associated with the meningeal blood vasculature in a mural location. They were variously dubbed mural LECs (muLECs used hereafter), brain LECs and fluorescent granular perithelial cells by the laboratories that described them (Bower et al., 2017; van Lessen et al., 2017; Venero Galanternik et al., 2017).

muLECs were found to be capable of scavenging wastes in the meninges, including large molecular weight fluorescent dyes, acetylated‐low density lipoprotein and even β‐amyloid protein introduced into the brain (Huisman et al., 2022; Jeong et al., 2021; Shibata‐Germanos et al., 2020). This scavenging capability was in part dependent on the mannose receptor and likely other scavenging receptors expressed by these cells at high levels, such as the Stabilin‐1 and Stabilin‐2 scavenger receptors (Huisman et al., 2022). As well as this remarkable waste clearance capability, muLECs were also found to produce vascular endothelial growth factors and to influence the formation of a normally patterned meningeal blood‐vessel network (Bower et al., 2017). Finally, a recent study has suggested that these cells may have an inherent plasticity and be capable of directly converting to blood vessels upon wounding of the brain, to contribute to the regenerative response (Chen et al., 2019). Although the functions of these cells are still being unraveled, it is clear that LECs found surrounding the larval midbrain in zebrafish undergo a remarkable and unexpected transition into individual scavenger cells that can clear waste and control local blood vessels. How evolutionarily conserved this transition and the function of these cells are remains to be fully understood.

## 12 A TRANSITION FROM LYMPHATIC VASCULAR TO BLOOD‐LIKE VASCULATURE

Another remarkable developmental transition that highlights the plasticity of LECs was recently reported by Das et al. (2022). A detailed serial imaging study of the vasculature of the anal fin revealed that lymphatic vessels initially vascularize the fin during larval and juvenile stages. These vessels originate from the thoracic duct and cardinal collateral lymphatic vessel and were lineage traced as they migrated ventrally, invaded the fin and formed a stereotypical network of lymphatics aligned along the fin rays. Surprisingly, after their initial formation, these lymphatics progressively lose their expression of LEC markers and gain expression of blood vascular marker genes (Figure 2c). This vessel network becomes connected to the blood vasculature and starts to carry blood with a low flow rate, also transitioning to a more blood vascular fate at a transcriptional level (Das et al., 2022). Overall, this is a process whereby a lymphatic vessel can form in development from a blood vessel, before it much later transdifferentiates back into a blood vessel or related blood‐carrying vessel (Figure 2c). This remarkable process was recapitulated in tissue regeneration following fin amputation and it is not just specific to the anal fin but also observed in the development of dorsal fin vasculature (Das et al., 2022).

Importantly, detailed anatomical studies have previously shown that Teleostei have a highly specialized, low blood flow vasculature that is connected to arteries and called secondary vasculature (Olson, 1996; Vogel & Claviez, 1981). These studies suggested that adult fish do not have lymphatic vasculature at all, but instead have a highly specialized secondary vasculature (Steffensen & Lomholt, 1992). Secondary vessels are characteristically devoid of blood flow under stable conditions but carry blood flow to tissues during exercise or in hypoxic conditions (Dahl Ejby Jensen et al., 2009; Steffensen et al., 1986; Vogel & Claviez, 1981). Previous work tracing these vessels in glass catfish with fluorescent dyes has demonstrated their distinctive anatomy, which is highly similar to the vessels now described (Dahl Ejby Jensen et al., 2009; Rummer et al., 2014). Furthermore, it has been noted that the transition from LEC to a blood carrying vasculature described in the fins (Das et al., 2022) may actually represent a transition of lymphatics to secondary vessels (Jeltsch & Alitalo, 2022). It is notable that the studies from expert anatomists have used mature adult animals to study physiological adaptations, while the studies from developmental biologists have examined the embryo to hunt for conserved aspects of embryogenesis. This fundamental difference in experimental approach likely underpins the current controversy; however, the findings of Das et al. (2022) may amalgamate the two models if the early lymphatics of the fish embryo are in fact antecedent of secondary vasculature.

It is appreciated that in some genetic knockout models (e.g. Prox1 knockout mice) LECs can transition to a blood vascular identity or even a hematopoietic lineage (Kazenwadel et al., 2023; Wigle & Oliver, 1999). This suggests that LEC plasticity can be harnessed to generate multiple cell types in experimental settings. The work highlighted above demonstrates that this plasticity has also been exploited throughout evolution, to produce blood vessels or secondary vasculature. It seems likely that as current studies shift from early developmental processes to the analysis of more physiologically adapted adult tissues, we may discover more examples of such LEC transitions, where plasticity is taken advantage of to generate new, highly specialized cell types.

## 13 CONCLUDING REMARKS

Transitions shape the zebrafish vasculature throughout its development. These include in the initial formation of the earliest blood vessels, transitions in cell states and cell mechanics within forming vessels, and transitions that diversify cell types and contribute organ‐specific vasculatures. This review has highlighted some key transitions and the associated molecular mechanisms that drive those transitions. It is notable that many of the pathways that drive these key developmental transitions go awry in vascular disease settings. For example, Notch signaling and BMP signaling drive key processes of primary and secondary angiogenesis (as described above) but these pathways are central in the pathogenesis of vascular anomalies and vascular malformations. Likewise, the genes that drive the transition from blood vasculature to lymphatic vasculature are regularly mutated in human lymphedema syndromes that occur due to deficiencies in this vasculature (for review, see Hogan & Schulte‐Merker, 2017; Oliver et al., 2020). These examples highlight that new understanding of fundamental transitions will inevitably enrich our understanding of the molecular drivers of pathogenesis and progression of vascular diseases.
